# Revisiting the Corneal and Blink Reflexes for Primary and Secondary Trigeminal Facial Pain Differentiation

**DOI:** 10.1155/2021/6664736

**Published:** 2021-02-09

**Authors:** Zafar Ali Khan

**Affiliations:** Department Oral & Maxillofacial Surgery, College of Dentistry, Jouf University, King Khalid Road, Sakaka, Al Jouf, Saudi Arabia

## Abstract

Trigeminal neuralgia is often misdiagnosed at initial presentation due to close connotation with dental pain and is often over diagnosed for the very same reasons leading to numerous unnecessary surgical procedures such as peripheral neurectomy and alcohol injections, while the actual cause may remain elusive for decades. Evaluation of the neurosensory system may disclose the correct anatomical location of the etiology. The neurological examination may be clouded by the sensory deficits subsequent to previous peripheral surgical procedures. The corneal and blink reflexes are integral measures of the trigeminal and facial neurosensory assessment, and their abnormal function may facilitate the identification of intrinsic disease of the brain stem. These reflexes can be employed to discover pathological lesions including intracranial space-occupying trigeminal, lateral medullary, cerebral hemispheric lesions, and degenerative diseases of the central nervous system. Dental surgeons and oral and maxillofacial surgeons should consider corneal reflex in neurological assessment of patient presenting with trigeminal neuralgia-like symptoms. Failure to evaluate corneal sensitivity may lead to delayed or inaccurate diagnosis and unsuitable or redundant treatment interventions. This simple noninvasive reflex can be performed by chair-side and may provide significant information regarding the origin of facial pain and is an invaluable part of clinical methods especially in remote and peripheral healthcare center practitioners where sophisticated radiographic investigations such as computed tomography and magnetic resonance imaging may not be available.

## 1. Introduction

The human eye is covered by a thin and transparent layer of tissue called cornea which contains the highest number of nerves in the whole body [1]. These nerves convey the touch, pain, and temperature sensations and perform a fundamental part in corneal reflexes [2]. The human cornea is three to six hundred times more sensitive than the skin with a density of seven thousand nociceptors per square millimeter approximately at the center. Unintentional eyelid shutting that can be induced by stimulating the corneal surface or by flickering direct light serve primarily as a shielding purpose constitute the corneal reflex. The blink reflex, on the other hand, essentially preserves the thin film of lacrimal fluid over the eye surface, occurring impromptu, or conversely is induced by various trigeminal or spinal stimulations [3].

**Figure 1:**
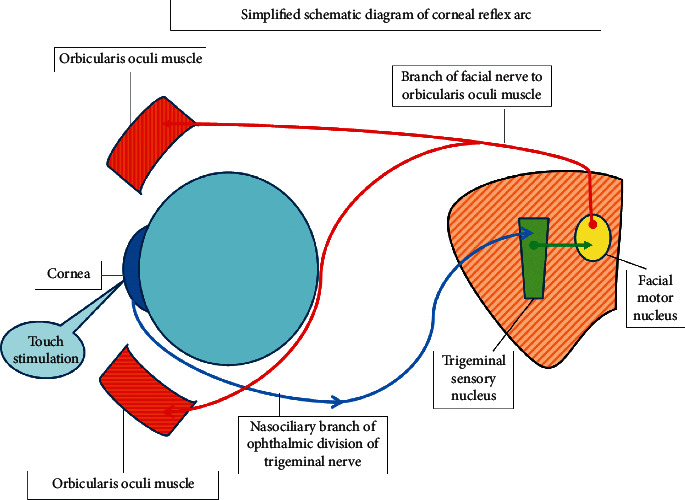
Simplified schematic diagram of corneal reflex arc.

Corneal and blinks reflexes have several common features, and each results in excitation of orbicularis oculi motor units and lid closure. The nasociliary and supraorbital branch of the ophthalmic division of the trigeminal nerve gives origin to the afferent innervation for the corneal and blink reflexes, respectively, while the efferent motor response is interceded via branch of the facial nerve to the orbicularis oculi muscle. Hence, these reflexes are essential instruments for assessment of the integrity of the trigeminal and facial cranial nerves which comprise the reflex arc [4] (Figure 1).

Trigeminal neuralgia, also known as tic douloureux, is a bursting painful condition that is characterized by agonizing, piercing paroxysms, inflicting one or more divisions of the trigeminal nerve, with less than 5% of the cases involving the ophthalmic division, while the mandibular division is affected in 70% of the cases. The attack can transpire suddenly as brief electric current-like contraction lingering for a few seconds to few minutes, or conversely, it is triggered by slight stimuli touching the facial skin including mild wind or even sound vibration rendering the patient unable to chew, eat, drink, shave, or brush their teeth for fear of impending attack. Trigeminal neuralgia is not characterized by objective sensory or motor deficits, but the patient may present with a subjective hypesthesias or numbness over the facial skin in the distribution of trigeminal nerve branches. The diagnosis is based on history alone; however, primary disorder must be differentiated from similar symptoms secondary to other more ominous causes. Documenting the age of commencement of symptoms is significant in such cases as the advent of trigeminal neuralgia in a young patient should raise the suspicion of secondary causes including multiple sclerosis or intracranial space-occupying lesions that may lead to compressive demyelination of the trigeminal root entry zone at the lateral pons [5]. Trigeminal neuralgia is often misdiagnosed at initial presentation due to close connotation with dental pain causing various unnecessary procedures directed to relieve the supposed dental origin of pain. Paradoxically, this disorder is often over diagnosed for the very same reasons leading to numerous unnecessary surgical procedures such as peripheral neurectomy and alcohol injections, while the actual cause of symptoms may remain elusive for many years from general clinicians. A meticulous and focused evaluation of the neurosensory system discloses the correct anatomical location of the correct etiology. The neurological examination may be clouded by the sensory deficits subsequent to peripheral surgical procedures performed in pursuit of providing long-lasting relief from primary trigeminal neuralgic symptoms [6, 7]. In this study, I have reviewed the role of the corneal and blink reflexes in differentiation and diagnosis of the primary idiopathic trigeminal neuralgia from the secondary neuralgia.

### 1.1. Role of Corneal and Blink Reflexes in Neurological Examination

The corneal and blink reflexes are not only integral measures of the trigeminal and facial neurosensory assessment, but the abnormal function may facilitate the identification of intrinsic disease of the brain stem as well. These reflexes can be employed to discover a range of different pathological lesions including intracranial space-occupying trigeminal, lateral medullary, cerebral hemispheric lesions, and degenerative diseases of the central nervous system [1, 4, 5] (Table 1).

### 1.2. Role in Localization of Trigeminal Nerve Lesions

**Figure 2:**
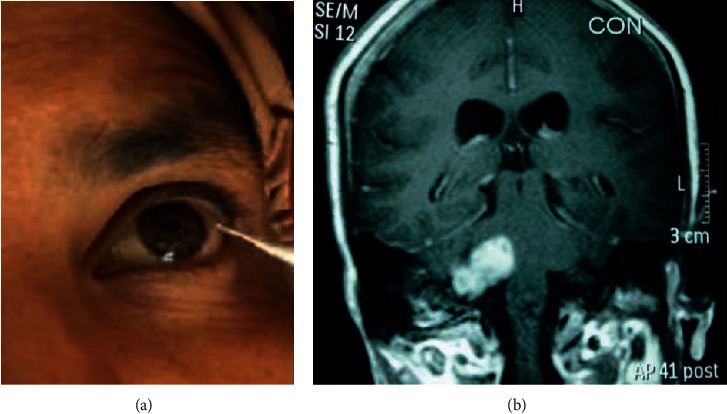
A 50-year-old female presenting with electric shock-like pain on the right side of the face for last 10 years. Patient had a history of multiple neurectomy of the infraorbital, mental, and inferior alveolar nerve with temporary relief followed by recurrence of symptoms. Corneal reflex was found absent. MRI revealed 2.2 × 2.2 × 2.7 extra-axial mass in the right cerebellopontine angle cistern suggestive of acoustic neuroma.

Measurements of delays in these reflexes have been reported as reliable in localization of supranuclear, nuclear, or peripheral nerve lesions. Trigeminal nerve may become compressed anywhere in the region brain-stem nuclei, the gasserian ganglion, or in the root entry zone at the cerebellopontine angle region and reveal symptoms of diminished sensations on the facial skin in association with hearing loss, facial muscle weakness, and complete loss or delay in reflex [4] (Figure 2).

The provoked reaction permits measurement of the delay in reflex after the stimulation of the afferent or the efferent nerve and noting the time taken by orbicularis oculi muscle contraction bilaterally [5].

### 1.3. Technique of Corneal Reflex

Lightly touching the surface of the cornea with a delicate material such as a cotton swab or wisp induces a rapid bilateral blink. Corneal reflex evaluation can be made while the patient looks to the side and the cornea is mechanically stimulated approaching from the temporal direction with a saline-soaked cotton tip or a droplet of saline or air ejected with an empty disposable syringe tip. The direct gaze on the oncoming object may cause the patient to blink in response to visual threat and may lead to misinterpretation of the reflex [6, 7]. The stimulus application to the corneal surface is fundamental to maximize the reflex yield. It has been reported that normal volunteers with healthy cornea can reliably distinguish the stimulus; nevertheless, the strength and sensitivity of corneal stimulation is considerably greater than the temporal conjunctiva [8]. The reflex is achieved preferably with approaching from the periphery to the middle portion of the cornea while avoiding the pupil and the field of vision in the center. It is preferable to use the noninjurious objects such as a slight saline jet emission from the syringe tip to prevent any chance of scratching the cornea during the process [9]. If gentle techniques fail to provoke the reflex, then the evaluator may continue with intensifying stimuli strength to acquire a conclusive response or confirm the lack of the response [10]. Slightly and steadily touching the cornea with a cotton-tipped applicator is considered the most effective method to achieve maximum stimulation of corneal nerve endings [4].

### 1.4. Technique for Blink Reflex

The blink reflex is considered the electronic equivalent of the corneal reflex that is utilized to serve the same diagnostic purpose. An electrical stimulus is applied to the supraorbital nerve, and evoked responses are recorded over various muscles innervated by the facial nerve. The blink reflex requires the electromyographic or nerve conduction study machine with at least two-channel recording capabilities and recording and dispersive electrodes [11]. The cathode (i.e., the negative electrode) of the transcutaneous electric nerve stimulator is placed exactly on the supraorbital notch region which indicates the path of the supraorbital branch of the ophthalmic division of the trigeminal nerve. The rest of the electrodes are placed on the face, with two on the inferior part of both orbicularis oculi just below the lower eyelids, while one electrode is placed on the zygomatic arches as reference. One dispersive electrode is placed either over the forehead or below the chin for prevention of any possible thermal injury to the underlying tissue. Transcutaneous electric stimulation of the supraorbital nerve elicits two responses in the orbicularis oculi muscles: the early (R1) component in the ipsilateral muscle and the late (R2) component bilaterally. The pattern of abnormal responses (early and late, direct and crossed) indicates which part of the reflex circuit is affected [12].

### 1.5. Significance in Trigeminal Neuralgia

All of the trigeminal reflexes and sensations of touch, two point discrimination, pressure, temperature, and pain are reported to be unaffected in classic or typical trigeminal neuralgia cases unlike the secondary type; therefore, the neurophysiologic examination and trigeminal reflex testing represents the paramount and the most valuable and dependable measure for the diagnosis and differentiation of primary and secondary TN [10]. This differentiation is important as the treatment is different in both cases and early identification can guide the clinician to correct and timely treatment options. TN patients are often subjected to unwarranted and repeated peripheral neurectomies and alcohol injections, and in such patients, if neurosensory evaluation reveals abnormal sensation or numbness over the face in the distribution of the trigeminal nerve region, it cannot be confirmed weather it is secondary to previous neurectomy and alcohol injection or a manifestation intracranial lesion or underlying the disease process. Corneal and blink reflex remains intact despite previous failed attempt for treatment of patient's symptoms by peripheral neuroablative procedures [13, 14]. The abnormalities found in these reflexes can facilitate in the diagnosis of various intracranial space-occupying lesions as discussed previously, which can be of utmost significance in early differentiation between primary idiopathic trigeminal neuralgia and secondary trigeminal neuralgia caused by space-occupying or vascular lesions of the cerebellopontine angle or inside the semilunar trigeminal ganglion that can mimic primary trigeminal neuralgia symptoms, and the pain in such situations is more enduring and assiduous accompanied with diminution or absence of corneal reflexes as a reliable sign pointing to a secondary cause of pain in such cases [5].

### 1.6. Conditions of Corneal Innervation and Sensation Alterations

There are various conditions and situations that may alter corneal innervation and sensation which may subsequently limit the utility of corneal reflex in differentiation of primary and secondary trigeminal neuralgia (Table 2).

### 1.7. Infection

Various infections including type 1 herpes simplex virus and varicella zoster virus, *Mycobacterium leprae*, and fungal infections can harm both the parenchyma and nerves of the cornea which subsequently alter the corneal reflex and limit its use in such situations.

### 1.8. Herpes Infection

Herpes zoster virus affecting the ophthalmic region is an agonizing and overwhelming situation arising due to reactivation of virus in the trigeminal ophthalmic division. Ophthalmic involvement may involve any portion between the conjunctiva and the optic nerve and is accompanied by a wide variety of inflammations causing ulceration and corneal perforation. Numerous research studies have observed the physical damage of nerves of the cornea due to infection of zoster virus, associated with reduced sensation which can consequently hamper the utilization of corneal reflex in postherpetic neuralgia cases [15]. Similarly, type I herpes simplex virus can also infect the ophthalmic region, and in such cases, changes below basal plexus has been reported in both eyes of patients that associate congruently with corneal sensation reduction related to length of the infection period and episodes of recurrences [2].

### 1.9. Leprosy

Leprosy has been reported to be associated with variations in nerve density of the stroma, abnormalities in epithelial nerves, and swelling, twisting, and convolution of the corneal nerve, supplemented by reduced sensations [16].

### 1.10. Corneal Transplant, Laser, and Other Ocular Surgeries

Several studies have reported a significant reduction in corneal sensation many years subsequent to transplantation. The most common corneal corrective surgical procedures include laser-assisted in-situ keratomileusis (LASIK) and photorefractive keratectomy (PRK) that utilize excimer photoablation for tissue removal. The magnitude of postsurgical corneal sensation reduction depends on the amount of tissue removal during the procedure [17, 18]. Laser panretinal photocoagulation for diabetic retinopathy or central retinal vein occlusion has also been reported to reduce sensations of the cornea [19].

### 1.11. Antiglaucoma Topical Medication

Reduction in sensation is documented in patients on glaucoma medication particularly with topical beta-adrenergic antagonists, specifically with a preservative benzalkonium chloride [20].

### 1.12. Advanced Age-Related Changes

Corneal sensation appears to decrease with age including thermal sensitivity to a cooling stimulus [21]. However, the significance of corneal reflex in diagnosis of trigeminal neuralgia due to intracranial lesions remains unaffected, as in most of the cases, the secondary trigeminal neuralgia presents at an earlier age and often before the age of forty years [14].

### 1.13. Diabetes Mellitus

Diabetic neuropathy involving the unmyelinated C and A delta-fibers contribute to paresthesias and may reduce corneal sensation in diabetics [22, 23]. This can limit the use of corneal reflex for differentiation in primary and secondary trigeminal neuralgia in diabetic patients.

### 1.14. Thyroid Gland Disease

Any thyroid gland dysfunction especially Graves' disease may present with thyroid-related ophthalmological pathology that may lead to abnormal function of corneal nerve and associated reflexes [24].

### 1.15. Sjögren's Syndrome

This condition can compromise corneal nerve function as it causes distortion of microscopic architecture of the nerve. However, there is disagreement in literature, whether this distortion leads to corneal sensation reduction or causes increased sensitivity, which may affect the validity of various testing modalities used to assess the corneal sensitivity [25–27].

## 2. Conclusion

As the trigeminal neuralgia pain appears to be originating from structures of the face and oral cavity, patient primarily pursue a general dentist for pain relief. When such patients finally present at tertiary care centers seeking relief of pain, they have already endured multiple dental procedures that have caused irreversible damage. In contrast, vast majority of dental practitioners lack knowledge of facial pain due to various other causes and have a tendency to over diagnose trigeminal neuralgia, being the only other cause of pain apart from toothache with which they are acquainted. For general dentists and maxillofacial surgeons, thorough history and meticulous clinical examination with a special emphasis on comprehensive appraisal of cranial nerves are indispensable in eluding erroneous diagnosis and inappropriate interventions in patients presenting with facial pain. Dental surgeons and oral and maxillofacial surgeons should consider corneal reflex in neurological assessment of patient presenting with trigeminal neuralgia-like symptoms. Failure to evaluate corneal sensitivity may lead to delayed or inaccurate diagnosis and unsuitable or redundant treatment interventions. This simple noninvasive reflex can be performed by chair-side and may provide significant information regarding the origin of facial pain and is an invaluable part of clinical methods especially in remote and peripheral healthcare center practitioners where sophisticated radiographic investigations such as computed tomography and magnetic resonance imaging may not be available. Its use may be limited in some local and systemic conditions of corneal hypoesthesia.

## Figures and Tables

**Table 1:** Lesions and conditions that can present with trigeminal neuralgia-like pain with loss of corneal reflex.

Viral infections	Herpes zoster
Intracranial space-occupying lesions	Meningioma, schwannoma, acoustic neuroma, and AV malformations epidermoid tumors/cyst
Demyelinating disorders	Multiple sclerosis
Inflammatory disorders	Tolosa–Hunt syndrome Gradenigo's syndrome
Others	Arnold–Chiari 1 malformation

**Table 2:** Situations of corneal sensation alterations that may limit the use of corneal reflex if present concurrently with trigeminal neuralgia.

Infectious diseases Type 1 herpes simplex Varicella zoster virus *Mycobacterium leprae* Fungal infections
Autoimmune disorder Diabetes mellitus Grave's disease Sjögren's syndrome
Ophthalmic procedures and surgeries Corneal transplant Laser and other ocular surgeries including laser-assisted in situ keratomileusis (LASIK) Photorefractive keratectomy (PRK)
Ophthalmic medication Antiglaucoma topical medication especially topical beta-adrenergic antagonists Benzalkonium chloride
Age Advanced age

## Data Availability

No data were used to support this study.
